# The Effect of Patient Factors and Cotreatments on the Magnitude of Potassium Lowering with Insulin–Glucose Treatment in Patients with Hyperkalemia

**DOI:** 10.3390/epidemiologia2010003

**Published:** 2021-01-11

**Authors:** Andy K. H. Lim, Ljiljana Crnobrnja, Manogna Metlapalli, Mauli Govinna, Cathy Jiang

**Affiliations:** 1 Department of General Medicine, Monash Health, Clayton, VIC 3168, Australia; ljiljana.crnobrnja@monashhealth.org (L.C.); manogna.metlapalli@monashhealth.org (M.M.); Mauli.govinna@monashhealth.org (M.G.); Cathy.jiang@monashhealth.org (C.J.); 2 Department of Medicine, School of Clinical Sciences, Monash University, Clayton, VIC 3168, Australia

**Keywords:** potassium, hyperkalemia, insulin–dextrose, insulin–glucose, sodium polystyrene sulfonate, salbutamol, sodium bicarbonate, chronic kidney disease, acute kidney injury, dialysis

## Abstract

The management of hyperkalemia with insulin–glucose/dextrose treatment (IDT) may be influenced by patient factors and cotreatments. We aimed to determine the magnitude of potassium lowering by IDT while considering patient factors and cotreatments. We observed the change in serum potassium in 410 patients with a mean serum potassium of 6.6 mmol/L (SD, 0.6 mmol/L) treated with IDT at three major metropolitan hospitals. Mean potassium lowering was 1.4 mmol/L (SD, 0.8 mmol/L) and 53% achieved normokalemia. Cotreatment with sodium polystyrene sulfonate, salbutamol, or sodium bicarbonate occurred in 64%, 12%, and 10% of patients, respectively. In multiple linear regression analysis, cotreatment with sodium polystyrene sulfonate or sodium bicarbonate was not associated with any significant reduction in serum potassium beyond that achieved by IDT, within the initial 6 h of treatment. We observed an additional lowering of serum potassium with salbutamol of 0.3 mmol/L (95% CI: 0.1 to 0.6 mmol/L; *p* = 0.009) but the clinical significance was unclear as the proportion of patients achieving normokalemia was not affected by cotreatment within the initial 6 h after IDT. We also found evidence that the potassium-lowering effect of IDT was dependent on the pre-treatment serum potassium. For every 1 mmol/L increase in pre-treatment serum potassium over 6.0 mmol/L, there was an associated 0.7 mmol/L increase in the potassium-lowering effect of IDT, on average, which was independent of any cotreatment. There was no significant impact of acute kidney injury or chronic kidney disease status on the efficacy of IDT.

## 1. Introduction

Severe hyperkalemia with a serum potassium (K^+^) of 6.0 mmol/L or higher is associated with an increased risk of cardiac arrhythmia and mortality [1]. Several treatments are available in the emergency setting for acute lowering of serum K^+^ to prevent cardiac arrhythmia. These include treatments which shift K^+^ intracellularly, such as intravenous insulin (with glucose or dextrose), nebulized salbutamol (albuterol), and sodium bicarbonate. In addition, enhanced elimination may be achieved by administering a cation exchange resin such as sodium polystyrene sulfonate (SPS) which promotes gastrointestinal excretion, but the action is often delayed and less predictable. Dialysis is another option for patients who have kidney failure or acute kidney injury (AKI) who have an inadequate response or are unlikely to respond to the other treatments [2].

Among the available treatment options, the most used and the preferred first-line treatment is 10 units of regular insulin given as an intravenous bolus with an intravenous bolus of 50 mL of 50% glucose or 50 mL of 50% dextrose (25 g of glucose as the active ingredient). Even though there have been variations to the insulin and glucose dose and method of delivery (bolus vs. infusion), none are convincingly superior to this basic approach [3]. Many of the early studies with insulin–glucose/dextrose treatment (IDT) were performed in patients with end-stage kidney disease who were dialysis dependent or had significant chronic kidney disease (CKD) [2,3]. In one systematic review of several small studies, the mean change in serum K^+^ one hour after 10 units of regular insulin administration was 0.8 mmol/L (standard deviation (SD), 0.3 mmol/L). Three of the five studies were conducted in patients on chronic hemodialysis, while the other two were conducted in patients with AKI or CKD [3]. Notably, patients who received any concurrent treatment for hyperkalemia were excluded in these studies.

It remains unclear if the K^+^ lowering effect of IDT is different between patients with AKI, CKD, or kidney failure on dialysis. Similarly, the effect of other agents, such as salbutamol and sodium bicarbonate, is less studied in non-dialysis patients. In one study, the addition of bicarbonate therapy to IDT or salbutamol did not enhance the K^+^-lowering effect of these treatments in hemodialysis patients [4], but it is not clear whether the same applies to non-dialysis patients. The aims of this study were to quantify the magnitude of the K^+^-lowering effect of IDT in a general population of patients (a mix of patients with normal renal function, AKI, CKD, and chronic dialysis) who required treatment for hyperkalemia, while taking into account the additional effect of patient factors, cotreatments, and baseline serum K^+^.

## 2. Methods

### 2.1. Study Design and Setting

This was a retrospective observational study conducted at three major metropolitan hospitals in the Monash Health hospital network in Victoria, Australia. The study population was all patients who were treated with IDT at any of the three sites from January 2019 to March 2020. This included patients in the emergency department, general wards, and intensive care unit.

### 2.2. Ethics Approval

This study was approved by the Monash Health Human Research Ethics Committee (Monash HREC reference, RES-20-0000-191Q; date of approval: 18 March 2020. Ethical Review Manager reference, ERM 62695). Individual patient informed consent was waived as this was a retrospective study utilizing data routinely collected during clinical care based on existing treatment protocols, and no additional information was sought from any patient.

### 2.3. Patient Selection

The ICD-10 diagnosis code for hyperkalemia was used to identify potentially eligible patients. Patients were eligible for inclusion if they were ≥18 years old, had a confirmed serum K^+^ of ≥6.0 mmol/L, and received standard IDT (intravenous bolus of 10 units of regular insulin and 50 mL of 50% glucose or 50% dextrose). Patients who received an insulin infusion were not eligible. To avoid correlated data, patients who were re-treated for hyperkalemia during a subsequent episode of care were excluded. Patients were also excluded if they died within 6 h of treatment, if there was insufficient biochemical monitoring to reliably determine the trough level of serum K^+^ (minimum requirement is ≥2 biochemistry tests within 6 h, with at least one within 2 h of IDT).

### 2.4. Primary Outcome

The extent of K^+^ lowering was assessed by calculating the (1) pre-treatment K^+^ *minus* post-treatment K^+^ (ΔK^+^), and (2) the proportion of patients achieving a normal serum K^+^ (<5.4 mmol/L). Thus, a positive value for ΔK^+^ denotes K^+^ lowering, and a negative value for ΔK^+^ denotes a K^+^ increase. In this study, the post-treatment K^+^ is the lowest K^+^ achieved within 6 h of IDT, which is the period of protocol monitoring.

### 2.5. Cotreatments, Variables, and Subgroups

The cotreatments in this study were SPS, mostly 30 g orally (range 15 to 60 g); salbutamol, mostly 10 mg nebulized (range, 5 to 20 mg); and sodium bicarbonate, 20 to 50 mmol by the oral or intravenous route. The subgroups of interest in this study were based on categories of kidney function: (1) preserved kidney function, eGFR ≥ 60 mL/min/1.73 m^2^, (2) CKD, eGFR < 60 mL/min/1.73 m^2^, (3) chronic dialysis, patients on maintenance dialysis irrespective of eGFR, and (4) AKI, as defined by the Kidney Disease Improving Global Outcomes (KDIGO) collaboration criteria, as an increase in serum creatinine by ≥26.5 µmol/L (≥0.3 mg/dL) within 48 h, or increase in serum creatinine to ≥1.5 times baseline which is known or presumed to have occurred within 7 days prior [5].

Body mass index (BMI) was determined by dividing weight (in kg) by height (in meters) squared, and a BMI ≥ 30 kg/m^2^ defined obese status. Lean body mass (LBM) was estimated with the Boer formula: LBM (male) = 0.407*weight* + 0.267*height −* 19.2; LBM (female) = 0.252*weight* + 0.473*height* − 48.3 [6]. A high risk of malnutrition was defined as a score of ≥2 using the Malnutrition Universal Screening Tool (MUST) [7]. The presence of sepsis was defined using the Sepsis-3 criteria [8]. We defined active malignancy as the presence of locally invasive or metastatic solid cancers, or hematological malignancy for which the patient is receiving active treatment with chemotherapy, hormonal therapy, or immune therapy.

### 2.6. Statistics

We used the chi-squared (*χ*^2^) statistic to test the association between two categorical variables. We compared the means of continuous variables between two groups with the *t*-test, or with one-way ANOVA for comparing multiple groups. The correlation between two continuous variables was determined by Pearson’s correlation coefficient (*r*). An *r* ≥ 0.5 was considered a moderate correlation, while an *r* ≥ 0.7 was considered a strong correlation. We used multiple linear regression (with the purposeful selection method) to model the effect of cotreatments on ΔK^+^, and the effect of CKD and AKI on ΔK^+^. In brief, all cotreatments were retained in the multivariable model as variables of primary interest in this study. Other variables with a *p* < 0.20 in the univariable analysis were added to the multivariable model. Through backward elimination, we retained variables with a *p* < 0.05 and variables which changed the *b* coefficient of the cotreatments by ≥10% if removed from the model. In the final multivariable model, we checked for statistical interaction between the covariates at the 1% level, and examined for collinearity by determining the variance inflation factor. The model diagnostics included an assessment of the linearity of continuous variables by fractional polynomials, assessment of model fit by residual analysis, and examination of the residual versus fitted and leverage plots for any outliers or influential points. All the analyses were performed with STATA 16 (StataCorp, College Station, TX, USA) and a *p* < 0.05 was considered statistically significant.

## 3. Results

### 3.1. Patient Characteristics

**Figure 1:**
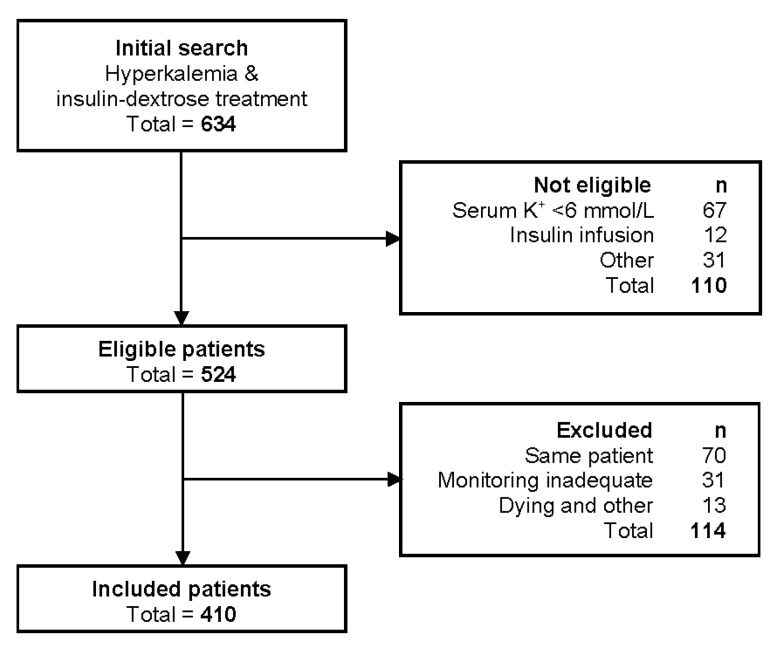
Study flow diagram demonstrating patient selection, inclusions, and exclusions.

The ICD-10 search for eligible patients and the reasons for exclusions are summarized in a flow diagram shown in Figure 1. The baseline characteristics of the patients who were included in the study are shown in Table 1. There was a male predominance in the study population. The average BMI of the patients was in the overweight category, and over one third were classified as obese. Diabetes mellitus and CKD were prevalent comorbidities, and 20% of patients were on long-term dialysis. Around half of all patients experienced AKI but stage 3 AKI (increase in serum creatinine ≥ 354 µmol/L, or ≥3.0 times baseline, or initiation of renal replacement therapy) only occurred in 13% of patients. Over one third of patients were treated with beta blockers or renin–angiotensin system (RAS) inhibitors, which are medications which could theoretically affect K^+^ homeostasis and the efficacy of IDT.

### 3.2. Cotreatments and ΔK^+^

From an average baseline serum K^+^ of 6.6 mmol/L, the overall mean ΔK^+^ was 1.4 mmol/L. The calculated ΔK^+^ showed a normal distribution. There was a very small difference in the baseline serum K^+^ between patients who received at least one cotreatment (mean, 6.65 mmol/L; 95% CI: 6.58 to 6.71 mmol/L) and those who only received IDT (mean, 6.52 mmol/L; 95% CI: 6.40 to 6.64 mmol/L), but this difference did not reach statistical significance (*t*_408_ = 1.89, *p* = 0.06). SPS was the most frequently used cotreatment, while salbutamol and sodium bicarbonate were infrequently encountered (Table 2). Overall, just over half of the patients achieved a nadir serum K^+^ within the normal reference range (<5.4 mmol/L) after treatment. Although 74% of patients had at least one cotreatment to lower serum K^+^, the cotreatments did not affect the mean ΔK^+^ or the proportion of patients who achieved normokalemia, as summarized in Table 2.

### 3.3. Effect of Cotreatments on ΔK^+^

To further evaluate the effect of cotreatment, we used multiple linear regression to model the effects of a cotreatment on ΔK^+^, adjusting for potential confounders and other cotreatments. From the initial univariable analysis (Supplementary Table S1), we identified the variables with a *p* < 0.20 as age, active malignancy, CKD, and cirrhosis. In the multivariable model, we retained all cotreatments and variables with a *p* < 0.05 or variables which changed the coefficients of the cotreatments by >10% (Table 3). When given with IDT, SPS did not lower serum K^+^ acutely, and the effect of salbutamol was modest, lowering serum K^+^ by around 0.3 mmol/L, on average, after allowing for the other covariates. The effect of sodium bicarbonate on serum K^+^ was also weak, and may not be clinically significant.

### 3.4. Effect of Peak Serum K^+^ on Efficacy of Insulin–Glucose/Dextrose Treatment

**Figure 2:**
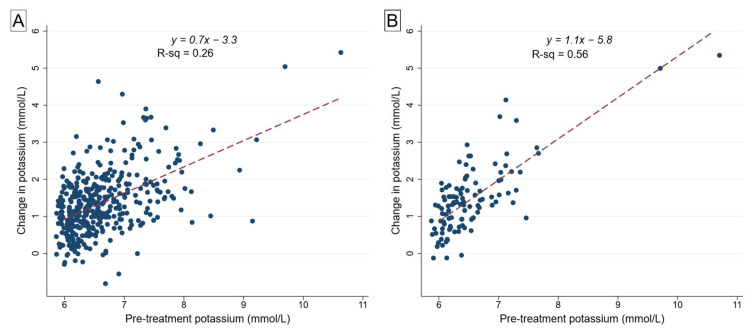
Scatter diagrams of the change in serum potassium (*y*) across the range of pre-treatment serum potassium levels (*x*), with a line of best fit for linear regression (dashed) for (**A**) all study patients irrespective of treatment received (*n* = 410); (**B**) patients who only received insulin–glucose/dextrose treatment (*n* = 109).

The effect of IDT on ΔK^+^ showed a significant positive correlation with the pre-treatment serum K^+^ level (*r* = 0.52, *p* < 0.001, for all patients; *r* = 0.75, *p* < 0.001, for patients who received IDT only). The higher the pre-treatment serum K^+^, the larger the ΔK^+^ after IDT, even after excluding patients who received cotreatments (Figure 2). Although the distribution of baseline serum K^+^ was slightly skewed, a fractional polynomial analysis suggested no significant difference in the deviance of a model using the log-transformed baseline serum K^+^ or other powers compared to a linear fit, and the residuals for simple linear regression were also normally distributed (Supplementary Figure S1). We estimated that, on average, for every 1.0 mmol/L increase in pre-treatment serum K^+^ above 6.0 mmol/L, there was an associated 0.7 mmol/L increase in ΔK^+^ with IDT. Thus, the K^+^-lowering effect of IDT is more pronounced in severe hyperkalemia compared to mild hyperkalemia.

We also conducted a sensitivity analysis by excluding two patients with a relatively high leverage on the regression diagnostic plots, which corresponded to the two cases where the baseline serum K^+^ was in a range infrequently observed (baseline K^+^ of 9.6 mmol/L and 10.6 mmol/L). With these cases removed, the coefficient for ΔK^+^ declined slightly to 0.63 (95% CI: 0.50 to 0.76), resulting in a regression equation of ΔK^+^ = 0.6 (baseline K^+^) − 2.8. In the linear regression model for patients who received IDT only (no cotreatments), the removal of these two cases produced a coefficient for ΔK^+^ of 1.19 (95% CI: 0.86 to 1.51), resulting in a regression equation of ΔK^+^ = 1.2(baseline K^+^) − 6.3.

### 3.5. Effect of Chronic Kidney Disease on ΔK^+^

A comparison of K^+^ biochemistry and cotreatments by CKD status is shown in Table 4. There was no significant difference in ΔK^+^ between patients with an eGFR < 60 mL/min/1.73 m^2^ and patients with an eGFR ≥ 60 mL/min/1.73 m^2^, with a *t*_408_ = 1.42, and *p* = 0.16. Similarly, there was no difference in the mean ΔK^+^ between patients without CKD, with CKD, and dialysis patients by ANOVA (*F*_2, 407_ = 1.75, *p* = 0.17). The multivariable linear regression model confirmed there was no significant effect of CKD status on ΔK^+^ after adjusting for cotreatments, age, cirrhosis, and active malignancy (*b* = −0.14, 95% CI: −0.32 to 0.03, *p* = 0.11).

### 3.6. Effect of Acute Kidney Injury on ΔK^+^

A comparison of K^+^ biochemistry and cotreatments by AKI status is shown in Table 5. After excluding patients on chronic dialysis where AKI cannot be determined, the mean ΔK^+^ in patients with AKI was not different to patients without AKI (mean difference, 0.1 mmol/L; *t*_326_ = 0.9, *p* = 0.37). With multivariable regression, we confirmed there was no significant effect of AKI on ΔK^+^ after adjusting for cotreatments, age, CKD, cirrhosis, and malignancy (*b* = 0.03, 95% CI: −0.15 to 0.21, *p* = 0.71). However, patients with AKI had a mean baseline K^+^ which was 0.2 mmol/L higher than patients without AKI (*t*_326_ = 2.52, *p* < 0.001). Patients with AKI also had a lower proportion achieving normokalemia than patients without AKI (*χ*^2^ = 4.67, *p* = 0.031).

## 4. Discussion

In this observational study of a mixed population of patients with various CKD stages and hyperkalemia (K^+^ ≥ 6.0 mmol/L) treated with IDT using an intravenous bolus strategy, we found that the mean K^+^ lowering with IDT was approximately 1.4 mmol/L, but there was no clear evidence that cotreatment with SPS or sodium bicarbonate added additional benefit in the initial 6 h. The additional effect of salbutamol was modest, and may have contributed to a further lowering of K^+^ by 0.3 mmol/L, on average, after adjusting for other cotreatments and confounders. We further note that the efficacy of IDT was dependent on the pre-treatment serum K^+^ levels, such that K^+^ lowering was greater in patients with a higher pre-treatment K^+^. We did not find evidence that the efficacy of IDT was significantly reduced in patients with AKI, CKD, or dialysis in a real-world clinical practice.

The magnitude of the K^+^ lowering effect of IDT estimated from published prospective studies has been highly variable due to the small sample sizes and heterogeneity in the study population, interventions, and the pre-treatment K^+^, such that a meta-analysis of the treatment effect has not been possible in systematic reviews of IDT [3,9,10]. The reported ΔK^+^ in prospective adult studies of IDT where pre-treatment K^+^ was ≥6.0 mmol/L and 10 units of insulin were administered, have ranged from 0.8 to 1.1 mmol/L [3]. However, the overall quality of the evidence from these small studies has been poor [10]. The estimated ΔK^+^ in our study is higher than these previous reports. The reason for this is not entirely clear but may reflect the impact of cotreatment with salbutamol, or concurrent treatment of the underlying acute medical condition responsible for the initial presentation which may have improved the metabolic status of some patients. Our study is consistent with previous reports that there is a small additive effect of salbutamol on the ΔK^+^. We did not find any additional benefit of sodium bicarbonate but many patients who received bicarbonate may have received a less than ideal dose. However, bicarbonate may also be less effective than IDT or salbutamol when the patient is not severely acidotic [10]. The lack of apparent effect of SPS on lowering K^+^ acutely is not surprising. The onset of the action of SPS is typically delayed by several hours and its K^+^-lowering effect may not be fully realized for 10 to 12 h, where a ΔK^+^ of 1 mmol/L can be expected after an oral dose of 30 g [11].

To our knowledge, the finding that the efficacy of IDT was dependent on pre-treatment K^+^ is a novel finding. We postulate this may be due to the larger transcellular gradient of K^+^, resulting in a greater intracellular shift when insulin triggers activity of the Na^+^-H^+^ antiporter on cell membranes, increasing sodium entry into cells and consequent activation of Na^+^-K^+^ ATPase which promotes the influx of K^+^ into cells. This is a rather reassuring finding for clinicians, that IDT remains quite effective even at significantly elevated levels of K^+^. Furthermore, we did not find that the efficacy of IDT was reduced in patients with AKI or CKD, even though previous studies have suggested that insulin resistance has been observed in critically ill patients with AKI [12] and patients with CKD [13]. Again, this is reassuring for clinicians.

There were several limitations of our study. Firstly, there was heterogeneity in cotreatment choice and dosing. Although we attempted to control for this by multivariable analysis, residual confounding cannot be excluded. We did not account for changes in the acid–base status of patients from baseline to the post-IDT period. The timing of K^+^ testing post-treatment was also not uniform between patients. After the nadir of serum K^+^ at 60 to 120 min, serum ΔK^+^ has been observed to begin rebounding up slightly at around 180 min [10]. Nonetheless, the ΔK^+^ in our study was larger than previously reported, suggesting that we did not miss the window for detecting the peak effect of treatment.

The results of our analysis could be useful for future research. We suggest that future studies or systematic reviews consider the baseline (pre-treatment) K^+^ when determining K^+^ lowering with IDT because of the potential impact of the baseline K^+^ on the ΔK^+^. Our multivariable analysis suggested that patients with active malignancy and patients with cirrhosis may have a lesser response to IDT, compared to patients without these morbidities. These two subgroups deserve further evaluation as potentially high-risk populations.

In conclusion, IDT appears to be more effective at higher levels of K^+^. IDT efficacy was not diminished in AKI or CKD, and the addition of salbutamol may provide a small additional benefit for K^+^ lowering, but the need for sodium bicarbonate should be individualized.

## Figures and Tables

**Table 1:** Baseline patient characteristics and medication use (*n* = 410).

Characteristic	Value
Age, mean (SD), years	69.6 (15.9)
Female, *n* (%)	156 (38.1)
BMI, mean (SD), kg/m^2^	28.4 (7.9)
Obese, BMI ≥ 30 kg/m^2^, *n* (%)	146 (35.6)
Estimated lean body mass, mean (SD), kg	54.6 (12.2)
Diabetes mellitus, *n* (%)	245 (60.0)
High risk of malnutrition, *n* (%)	71 (17.3)
Chronic kidney disease, *n* (%) ^1^	288 (70.2)
Chronic dialysis, *n* (%)	82 (20.0)
Acute kidney injury, *n* (%) ^2^	206 (50.2)
Stage 1	86 (21.0)
Stage 2	68 (16.6)
Stage 3	52 (12.7)
Sepsis, *n* (%)	48 (11.7)
Active malignancy, *n* (%)	66 (16.1)
Oral hypoglycemics, *n* (%)	123 (30.0)
Baseline insulin treatment, *n* (%)	96 (23.4)
Beta blockers, *n* (%)	162 (39.5)
RAS blocker, *n* (%)	136 (33.2)

^1^ Estimated glomerular filtration rate < 60 mL/min/1.73 m^2^ and includes patients on dialysis. ^2^ Acute kidney injury staging based on the Kidney Disease Improving Global Outcomes criteria [5]. Abbreviations: BMI, body mass index; RAS, renin–angiotensin system.

**Table 2:** Potassium biochemistry and cotreatments for hyperkalemia by cotreatment status.

Characteristic	All Patients *n* = 410	IDT Only *n* = 109
Pre-treatment K^+^, mean (SD) mmol/L	6.6 (0.6)	6.5 (0.6)
Post-treatment K^+^, mean (SD) mmol/L	5.3 (0.7)	5.1 (0.6)
ΔK^+^, mean (SD) mmol/L	1.4 (0.8)	1.4 (0.9)
ΔK^+^, median (IQR) mmol/L	1.2 (0.8 to 1.8)	1.3 (0.8 to 1.8)
Normalization of K^+^, *n* (%)	219 (53.4)	69 (63.3)
Any cotreatment, *n* (%)	300 (74.3)	
Sodium polystyrene sulfonate, *n* (%)	264 (64.4)	
Salbutamol, *n* (%)	49 (12.0)	
Sodium bicarbonate, *n* (%)	39 (9.5)	

Abbreviation: IDT, insulin–glucose/dextrose treatment; ΔK^+^, change in serum potassium.

**Table 3:** Multiple regression of the change in potassium with insulin–glucose/dextrose cotreatments (*n* = 410).

Cotreatment	Univariable		Multivariable ^1^	
	Coef. (95% CI)	*p* Value	Coef. (95% CI)	*p* Value
SPS	−0.20 (−0.37 to −0.03)	0.020	−0.15 (−0.32 to 0.02)	0.087
Salbutamol	0.39 (0.14 to 0.64)	0.002	0.33 (0.08 to 0.57)	0.009
Sodium bicarbonate	0.27 (−0.01 to 0.55)	0.060	0.26 (−0.02 to 0.53)	0.064

^1^ Adjusted for cotreatments, age, cirrhosis, active malignancy, and chronic kidney disease. Abbreviations: SPS, sodium polystyrene sulfonate.

**Table 4:** Potassium biochemistry and cotreatments for hyperkalemia by chronic kidney disease status.

Characteristic	eGFR ≥ 60 *n* = 122	eGFR < 60 *n* = 206	Dialysis *n* = 82
Pre-treatment K^+^, mean (SD) mmol/L	6.6 (0.6)	6.6 (0.6)	6.7 (0.6)
Post-treatment K^+^, mean (SD) mmol/L	5.1 (0.8)	5.3 (0.6)	5.3 (0.9)
ΔK^+^, mean (SD) mmol/L	1.4 (0.9)	1.3 (0.8)	1.4 (0.9)
ΔK^+^, median (IQR) mmol/L	1.3 (0.8 to 1.9)	1.1 (0.8 to 1.6)	1.4 (0.7 to 1.9)
Normalization of K^+^, *n* (%)	74 (60.7)	101 (49.0)	44 (53.6)
Any cotreatment, *n* (%)	84 (68.9)	158 (76.7)	59 (72.0)
Sodium polystyrene sulfonate, *n* (%)	69 (56.6)	145 (70.4)	50 (61.0)
Salbutamol, *n* (%)	17 (13.9)	24 (11.7)	8 (9.8)
Sodium bicarbonate, *n* (%)	11 (9.0)	20 (9.7)	8 (9.8)

Abbreviation: eGFR, estimated glomerular filtration rate (ml/min/1.73 m^2^); ΔK^+^, change in serum potassium.

**Table 5:** Potassium biochemistry and cotreatments for hyperkalemia by acute kidney injury status.

Characteristic	No AKI ^1^ *n* = 132	AKI *n* = 196
Pre-treatment K^+^, mean (SD) mmol/L	6.4 (0.4)	6.7 (0.7)
Post-treatment K^+^, mean (SD) mmol/L	5.1 (0.6)	5.3 (0.7)
ΔK^+^, mean (SD) mmol/L	1.3 (0.7)	1.4 (0.9)
ΔK^+^, median (IQR) mmol/L	1.1 (0.8 to 1.7)	1.3 (0.8 to 1.8)
Normalization of K^+^, *n* (%)	80 (60.6)	95 (48.5)
Any cotreatment, *n* (%)	94 (71.2)	148 (75.5)
Sodium polystyrene sulfonate, *n* (%)	85 (64.4)	129 (65.8)
Salbutamol, *n* (%)	15 (11.4)	26 (13.3)
Sodium bicarbonate, *n* (%)	8 (6.1)	23 (11.7)

^1^ Dialysis patients excluded. Abbreviation: AKI, acute kidney injury; ΔK^+^, change in serum potassium.

## Data Availability

The data presented in this study are available on reasonable request from the corresponding author, subject to approval by the Monash Health Research Directorate.

## Supplementary Materials

The following are available online at https://www.mdpi.com/2673-3986/2/1/3/s1, Table S1: Univariable linear regression of potassium lowering on independent variables, Figure S1: Distribution of residuals from linear regression of the change in serum K^+^ after insulin–glucose/dextrose treatment on baseline serum K^+^.

## References

[1] Rossignol P., Legrand M., Kosiborod M., Hollenberg S.M., Peacock W.F., Emmett M., Epstein M., Kovesdy C.P., Yilmaz M.B., Stough W.G. (2016). Emergency management of severe hyperkalemia: Guideline for best practice and opportunities for the future. Pharmacol. Res..

[2] Sterns R.H., Grieff M., Bernstein P.L. (2016). Treatment of hyperkalemia: Something old, something new. Kidney Int..

[3] Harel Z., Kamel K.S. (2016). Optimal Dose and Method of Administration of Intravenous Insulin in the Management of Emergency Hyperkalemia: A Systematic Review. PLoS ONE.

[4] Allon M., Shanklin N. (1996). Effect of bicarbonate administration on plasma potassium in dialysis patients: Interactions with insulin and albuterol. Am. J. Kidney Dis..

[5] Kellum J.A., Lameire N., Aspelin P., Barsoum R.S., Burdmann E.A., Goldstein S.L., Herzog C.A., Joannidis M., Kribben A., Levey A.S. (2012). Kidney disease: Improving global outcomes (KDIGO) acute kidney injury work group. KDIGO clinical practice guideline for acute kidney injury. Kidney Int. Suppl..

[6] Boer P. (1984). Estimated lean body mass as an index for normalization of body fluid volumes in man. Am. J. Physiol..

[7] Stratton R.J., Hackston A., Longmore D., Dixon R., Price S., Stroud M., King C., Elia M. (2004). Malnutrition in hospital outpatients and inpatients: Prevalence, concurrent validity and ease of use of the ‘malnutrition universal screening tool’ (‘MUST’) for adults. Br. J. Nutr..

[8] Singer M., Deutschman C.S., Seymour C.W., Shankar-Hari M., Annane D., Bauer M., Bellomo R., Bernard G.R., Chiche J.D., Coopersmith C.M. (2016). The Third International Consensus Definitions for Sepsis and Septic Shock (Sepsis-3). JAMA.

[9] Varallo F.R., Trombotto V., Lucchetta R.C., Mastroianni P.C. (2019). Efficacy and safety of the pharmacotherapy used in the management of hyperkalemia: A systematic review. Pharm. Pract. (Granada).

[10] Batterink J., Cessford T.A., Taylor R.A.I. (2015). Pharmacological interventions for the acute management of hyperkalaemia in adults. Cochrane Database Syst. Rev..

[11] Kessler C., Ng J., Valdez K., Xie H., Geiger B. (2011). The use of sodium polystyrene sulfonate in the inpatient management of hyperkalemia. J. Hosp. Med..

[12] Basi S., Pupim L.B., Simmons E.M., Sezer M.T., Shyr Y., Freedman S., Chertow G.M., Mehta R.L., Paganini E., Himmelfarb J. (2005). Insulin resistance in critically ill patients with acute renal failure. Am. J. Physiol. Renal Physiol..

[13] Leyking S., Fliser D. (2014). Insulin resistance in CKD. Clin. J. Am. Soc. Nephrol..

