# Supplementary material for: The Effect of Patient Factors and Cotreatments on the Magnitude of Potassium Lowering with Insulin–Glucose Treatment in Patients with Hyperkalemia
Source: Epidemiologia (Basel). 2021 Jan 11;2(1):27–35. doi: 10.3390/epidemiologia2010003 (PMC9620900; doi:10.3390/epidemiologia2010003)
Supplement: Supplementary file 1 [file epidemiologia-02-00003-s001.pdf]

Supplementary Materials:

Table S1. Univariable linear regression of potassium lowering on independent variables (n = 410).

Parameter	Coefficient (95% C.I.)	p Value
Age, per 20-year increase	−0.11 (−0.21 to −0.01)	0.036
Female sex	0.00 (−0.17 to 0.17)	0.98
Obese (BMI >30 kg/m^2)	0.04 (−0.13 to 0.21)	0.67
Lean body mass, per 5 kg increase [1]	−0.00 (−0.02 to 0.01)	0.75
Diabetes mellitus	−0.04 (−0.21 to 0.13)	0.64
Active malignancy	−0.25 (−0.48 to −0.03)	0.024
High risk of malnutrition	0.03 (−0.19 to 0.325)	0.79
Chronic kidney disease	-0.13 (−0.31 to 0.05)	0.16
Chronic dialysis	0.30 (−0.04 to 0.64)	0.089
Acute kidney injury	0.07 (−0.09 to 0.24)	0.39
Cirrhosis	−0.56 (−0.92 to −0.20)	0.002
Beta-blocker use	0.08 (−0.09 to 0.24)	0.37
RAS blocker use	−0.01 (−0.18 to 0.16)	0.91

[1] Estimated by the Boer formula. Abbreviations: BMI, body mass index; RAS, renin-angiotensin system.

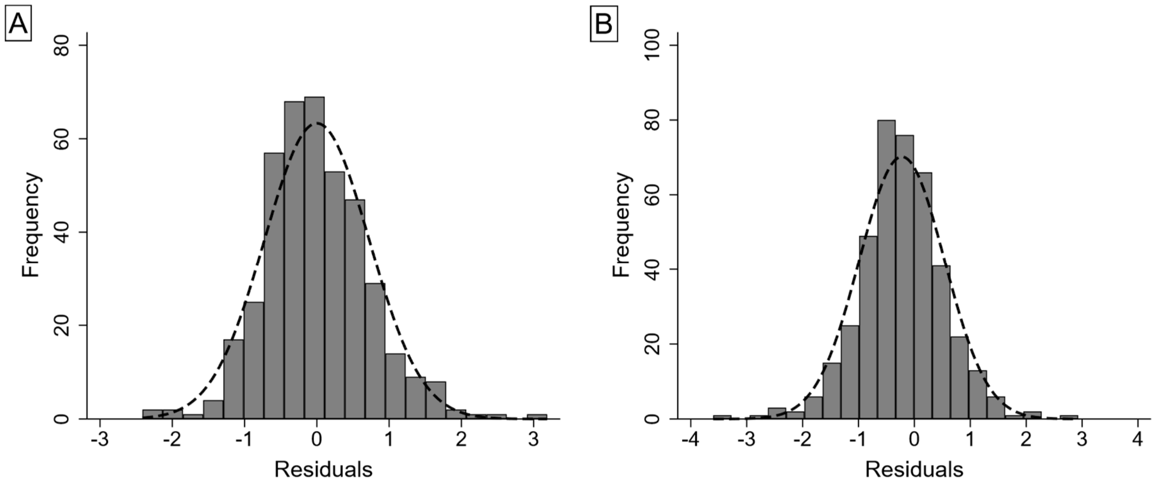

Figure S1. Histograms showing the (**A**) distribution of residuals from linear regression of the change in serum K+ (mmol/L) after insulin-glucose/dextrose treatment on baseline serum K+ for all patients (n = 410; regression equation, $y = 0.7x - 3.3$), and (**B**) for patients treated only with insulin–glucose/dextrose without any cotreatments (n = 109; regression equation, $y = 1.1x - 5.8$), with a normal distribution curve superimposed (dashed line).
